# Effects of High-Intensity Interval Training and Moderate-Intensity Continuous Training on Insulin Resistance Markers in Overweight and Obese Moroccan Adolescents

**DOI:** 10.3390/sports14080318

**Published:** 2026-07-29

**Authors:** Hamid Messaoudi, El Mokhtar El Ouali, Miloud Chakit, Bilel Cherif, Jihan Kartibou, Rachid Boujdi, Redouane Sarakh, Ismail Bouzekraoui Alaoui, Hervé Mayode, Omar Akhouayri

**Affiliations:** 1 Biology and Health Laboratory, Faculty of Sciences, Ibn Tofail University, Kenitra 14000, Morocco; jihan.kartibou@uit.ac.ma (J.K.); omar.akhouayri@uit.ac.ma (O.A.); 2 Sports Science Research Team, Institute of Sports Sciences, Hassan 1st University, Settat 26000, Morocco; 3 National School of Public Health, Rabat 1000, Morocco; miloud.chakit@uit.ac.ma; 4 HIGHT Sports, A HIGHT Group Company, Dubai, United Arab Emirates; bcherif@hgvcapital.com (B.C.); rsarakh@hgvcapital.com (R.S.); hmayode@hgvcapital.com (H.M.); 5 Higher School of Education and Training (ESEF), Ibn Tofail University, Kenitra 14000, Morocco; rachid.boujdi@uit.ac.ma; 6 Interdisciplinary Laboratory of Sport Sciences, Institute of Sport Professions, Ibn Tofail University, Kenitra 14000, Morocco; alaoui.ismail.bz@gmail.com

**Keywords:** exercise training, insulin resistance, HOMA-IR, overweight and obesity, adolescents

## Abstract

Previous studies have shown that physical activity (PA) can improve insulin sensitivity and reduce insulin resistance (IR), a key determinant of metabolic dysfunction. While the beneficial effects of PA are well-documented in adults, evidence regarding the relationship between PA intensity and insulin resistance in adolescents, particularly in North African populations, remains scarce. This study aimed to compare the effects of a 12-week school-based high-intensity interval training (HIIT) and moderate-intensity continuous training (MICT) intervention, versus usual physical education, on insulin resistance as assessed by the homeostasis model assessment of insulin resistance (HOMA-IR) in overweight and obese non-diabetic Moroccan adolescents. The study included 90 non-diabetic overweight and obese Moroccan adolescents aged 12 to 17 years. Participants were randomly assigned to three groups (HIIT, MICT, and control) within a 12-week school-based intervention. Insulin resistance was assessed using the HOMA-IR index. Linear regression models were used to examine the associations between exercise modality and HOMA-IR, adjusting for age, sex, baseline body mass index (BMI), and baseline HOMA-IR. Compared with the control group, both HIIT and MICT were associated with significant reductions in HOMA-IR. In the fully adjusted models, HIIT showed a greater decrease of −0.90 (95% CI: −1.07 to −0.72) (all *p* < 0.001) and MICT showed a decrease of −0.77 (95% CI: −0.94 to −0.61). Stratified analyses by BMI categories showed consistent improvements in insulin resistance for both overweight and obese adolescents. These findings suggest that school-based structured exercise interventions, particularly HIIT and MICT, independent of dietary modification, may contribute to improvements in insulin sensitivity and help reduce early metabolic risk in overweight and obese non-diabetic Moroccan adolescents. However, these results should be interpreted with caution given that dietary intake and other lifestyle factors were not strictly controlled.

## 1. Introduction

Over the past decades, overweight and obesity among children and adolescents have emerged as major global public health concerns. According to recent estimates from the World Health Organization (WHO), the prevalence of overweight and obesity in individuals aged 5 to 19 years has increased more than fourfold since 1975, reaching over 340 million worldwide in 2016 [1]. This upward trend is particularly pronounced in low- and middle-income countries undergoing rapid nutritional and lifestyle transitions. In North Africa, including Morocco, several national and regional reports have documented a steady increase in the prevalence of overweight and obesity among adolescents, occurring alongside marked changes in lifestyle patterns characterized by higher consumption of energy-dense foods, reduced levels of physical activity, and increasing sedentary behaviors [2]. These changes during adolescence, a critical developmental period, are of particular concern, as excess adiposity at this age has been shown to strongly track into adulthood and is associated with a sustained increase in cardiometabolic risk later in life [3,4].

Building on these concerns, adolescence represents a particularly critical developmental window in this context. Unlike in younger children, the convergence of hormonal, behavioral, and lifestyle changes during this period creates a unique vulnerability to early metabolic dysregulation, making this age group a priority target for preventive strategies [5,6]. Puberty-related hormonal changes transiently reduce insulin sensitivity in all adolescents; however, excess adiposity, particularly visceral fat accumulation, exacerbates this physiological reduction and promotes a chronic state of insulin resistance. Beyond these metabolic vulnerabilities, childhood obesity frequently predisposes youth to musculoskeletal constraints and common postural deviations, notably lumbar hyperlordosis, genu valgum, and flat feet (pes planus), which can alter biomechanics and further discourage participation in regular physical activities [7].

Adolescent obesity is associated with a wide range of metabolic complications, among which insulin resistance (IR) represents a key early pathophysiological alteration. Insulin resistance is defined as a reduced cellular responsiveness to the action of insulin, leading to impaired glucose uptake and compensatory hyperinsulinemia [8,9]. Persistent IR constitutes a central component of the metabolic syndrome and is strongly implicated in the development of type 2 diabetes mellitus (T2DM), cardiovascular disease, and non-alcoholic fatty liver disease (NAFLD). Globally, T2DM represents a major health burden. in 2021, an estimated 537 million adults were living with diabetes, accounting for 6.7 million deaths and approximately USD 966 billion in healthcare expenditures, figures projected to rise substantially by 2045 [10]. Although these estimates primarily concern adults, accumulating evidence indicates that the metabolic origins of T2DM often begin during childhood and adolescence, highlighting the importance of early prevention strategies [11].

Regular physical activity (PA) is widely recognized as a cornerstone of obesity prevention and metabolic health across the lifespan [12,13]. PA improves body composition by reducing fat mass, enhances cardiorespiratory fitness, and exerts favorable effects on glucose metabolism and insulin sensitivity through multiple mechanisms, including increased skeletal muscle glucose uptake, improved mitochondrial function, and reduced systemic inflammation [14]. In adolescents with overweight or obesity, structured exercise interventions have been shown to lower fasting insulin levels and improve indices of insulin resistance, even in the absence of substantial weight loss. Meta-analyses in pediatric populations consistently report reductions in HOMA-IR following moderate-to-vigorous physical activity programs, underscoring the role of exercise as a non-pharmacological strategy to counteract early metabolic dysfunction [15,16]. Despite this evidence, the magnitude of metabolic benefits appears to depend on the type, intensity, and organization of the exercise performed.

Among structured exercise modalities, HIIT and MICT have received increasing attention in pediatric and adolescent populations. HIIT is characterized by repeated short bouts of high-intensity exercise (≥90–100% of maximal aerobic capacity) interspersed with periods of active or passive recovery. This modality has gained popularity due to its time efficiency and its capacity to induce rapid metabolic adaptations, including enhanced insulin signaling, increased GLUT-4 expression, and improved mitochondrial oxidative capacity. In contrast, MICT typically consists of prolonged continuous exercise performed at a moderate intensity, usually corresponding to 60–80% of maximal aerobic capacity, and has long been recommended for improving aerobic fitness and metabolic health. In adults and, more recently, in adolescents with overweight or obesity, both HIIT and MICT have been associated with improvements in insulin resistance; however, findings remain heterogeneous, and the relative superiority of one modality over the other remains debated [17,18].

Despite growing interest in exercise-based interventions, several gaps persist in the literature. Few studies have directly compared the effects of HIIT and MICT on insulin resistance using standardized biomarkers, such as the Homeostasis Model Assessment of Insulin Resistance (HOMA-IR), in adolescents with overweight or obesity. Moreover, school-based intervention data from low- and middle-income countries, including North African contexts, remain scarce, and results across studies are sometimes contradictory due to differences in study design, intervention duration, and participant characteristics. In this context, the present study aimed to investigate the effects of a 12-week school-based HIIT and MICT intervention on insulin resistance, assessed by HOMA-IR, in overweight and obese Moroccan adolescents. By directly comparing these two exercise modalities, this study seeks to contribute to a better understanding of the most effective exercise strategies for improving early metabolic risk in this population.

## 2. Materials and Methods

### 2.1. Ethics and Informed Consent

Prior to participation, students and their parents or legal guardians received written and oral information about the study procedures. Written informed consent was obtained from all participants and from parents or legal guardians for those under 18 years of age, and written assent was obtained from all adolescents. This study was not registered in a public clinical trial registry, as it involved a structured physical education intervention without any medical treatment or pharmacological agent, and therefore does not meet the ICMJE criteria for mandatory prospective registration. The study protocol was reviewed and approved by the Institutional Review Board (Protocol code: LBS-2023-01) in accordance with the ethical standards for research involving minors.

### 2.2. Study Design

This study examined the effects of a 12-week (3-month) exercise training program, including HIIT and MICT, on insulin resistance and weight status in overweight and obese adolescents, assessed using the HOMA-IR index. The intervention was conducted between October 2023 and June 2024 at the Rabat Regional Academy of Education, Morocco. A total of 90 adolescents (45 boys and 45 girls), aged 12 to 17 years, were recruited from public and private middle and high schools, with mean ages of 14.2 ± 0.3 years for boys and 13.8 ± 0.2 years for girls.

### 2.3. Inclusion and Exclusion Criteria of Participants

Participants were included if they met the following criteria: (1) were aged between 12 and 17 years; (2) had overweight or obesity defined according to age- and sex-specific body mass index (BMI) percentiles based on World Health Organization (WHO) growth references (overweight: BMI-for-age ≥ 85th percentile; obesity: BMI-for-age ≥ 97th percentile); (3) were free from medical conditions or medications that could interfere with the study protocol (including cardiovascular disease, diabetes, orthopedic, neuromuscular, or neurological disorders); and (4) had no contraindication to physical activity. Exclusion criteria included the presence of psychiatric disorders, use of medications affecting the central nervous system (e.g., antidepressants), or failure to provide written informed consent.

### 2.4. Procedures

**Figure 1:**
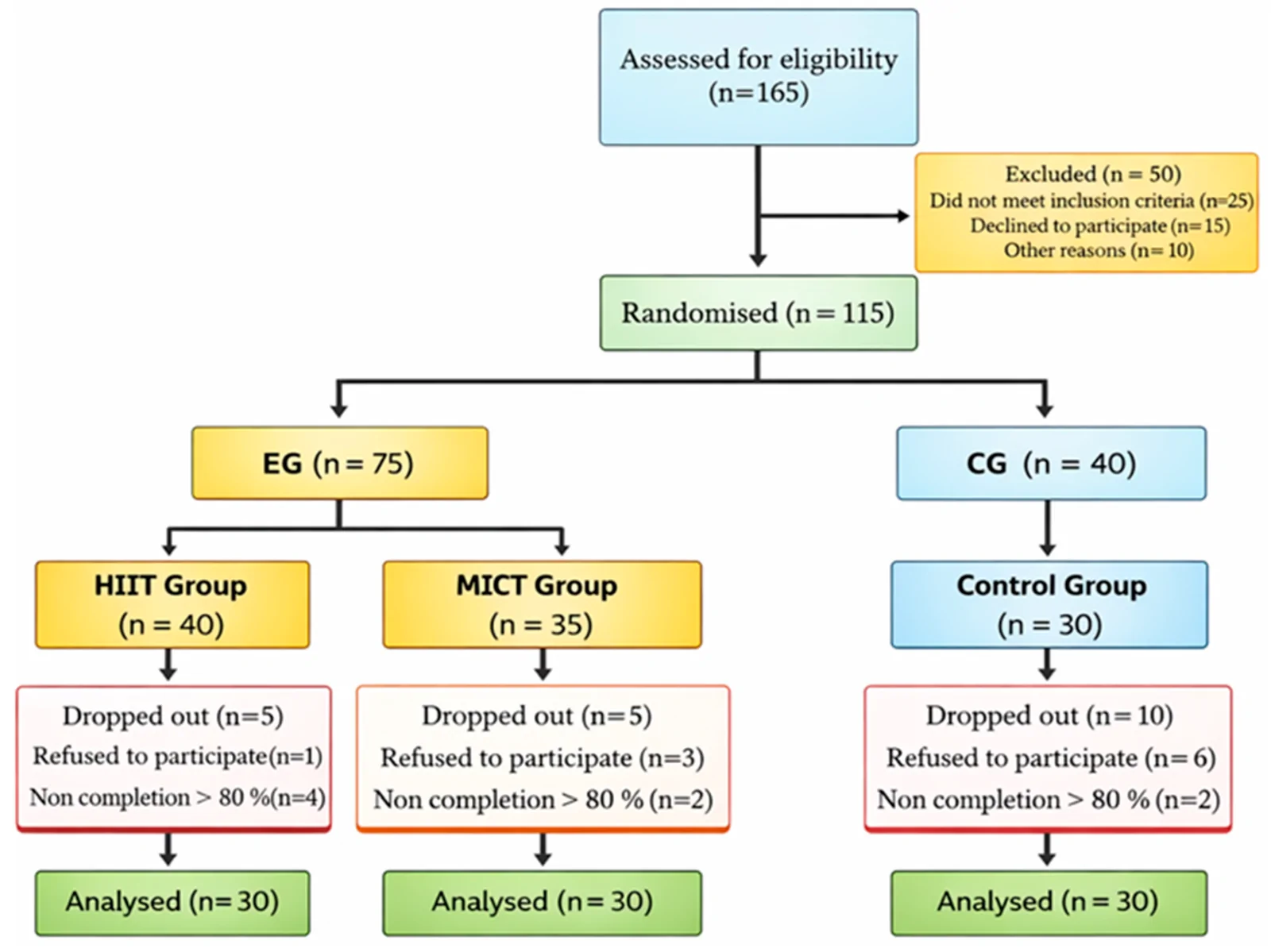
Flow chart of the study.

A total of 90 adolescents (45 girls and 45 boys), aged 12 to 17 years, were recruited for this study, an age range chosen due to its developmental importance, corresponding to the transition from childhood to adolescence and its consistency with previous research. The sample size was determined through a priori power analysis using GPower 3.1 for a repeated-measures ANOVA design (Time × Group interaction), with a significance level of α = 0.05, a medium effect size of f = 0.25, a statistical power of 1 − β = 0.95, a correlation among repeated measures of r = 0.50, and a nonsphericity correction of ε = 1.0, yielding a minimum required sample of 72 participants [19]. To account for a 20% attrition rate, the target sample was set at 90 participants (*n* = 30 per group) [N target = 72/(1 − 0.20) = 90]. Initial screening was conducted across all available school classes using BMI-for-age percentiles; 160 adolescents were identified with a BMI at or above the 85th percentile. Following application of exclusion criteria, including medical exemptions from Physical Education, pre-existing diseases, and other predefined criteria, 70 participants were excluded, resulting in the final sample of 90 participants. Recruitment took place in several schools (middle and high schools) reflecting a diversity of socioeconomic profiles. Participant recruitment and allocation are illustrated in the CONSORT flow diagram shown in Figure 1. Participants were randomly assigned to three groups: a HIIT group, MICT group and a control group that did not participate in any specific training program. All participants were instructed to maintain their usual lifestyle habits and to refrain from engaging in any additional physical activities outside of school during the study period. However, it should be noted that dietary intake was not formally controlled; no formal dietary assessment or nutritional counseling was implemented, and participants were not required to follow a specific diet. Furthermore, adherence to the restriction on outside physical activity was not objectively monitored using accelerometry or activity logs, which represents a limitation of the present study. All measurements and training sessions were supervised by the same physical education teachers and medical staff. Adherence to the exercise program was systematically monitored via school attendance registers managed by the supervising Physical Education teachers; all 90 participants met the minimum attendance threshold of 90% of sessions and were retained in the final analysis. To examine the impact of the intervention on insulin resistance, BMI, fasting glucose, fasting insulin, and the HOMA-IR index were measured before and after the training period.

### 2.5. Physical Exercise Program

The training intervention was conducted over a 12-week period, a duration consistent with the most commonly used and effective intervention length reported in the literature for inducing significant improvements in insulin resistance markers in adolescents with overweight or obesity [16,20], and was structured into three successive phases (weeks 1–4, weeks 5–8, and weeks 9–12), with a progressive increase in exercise intensity for both training programs. Training sessions were performed three times per week (Monday, Wednesday, and Friday) within the school setting and were supervised by qualified specialist teachers in physical education and sports. Attendance was systematically recorded at each session by the supervising Physical Education teachers via school attendance registers. All 90 participants attended at least 90% of the scheduled training sessions throughout the 12-week intervention period. Training intensity adherence was further ensured through continuous heart rate monitoring during each session.

The HIIT program consisted of repeated bouts of 15 s of exercise followed by 15 s of recovery throughout the entire intervention. Training intensity increased progressively from 90% of maximal aerobic speed (MAS) during weeks 1–4, to 95% MAS in weeks 5–8 and reached 100% MAS in weeks 9–12. Each session included eight series, with a total exercise duration ranging from 11 to 15 min.

**Figure 2:**
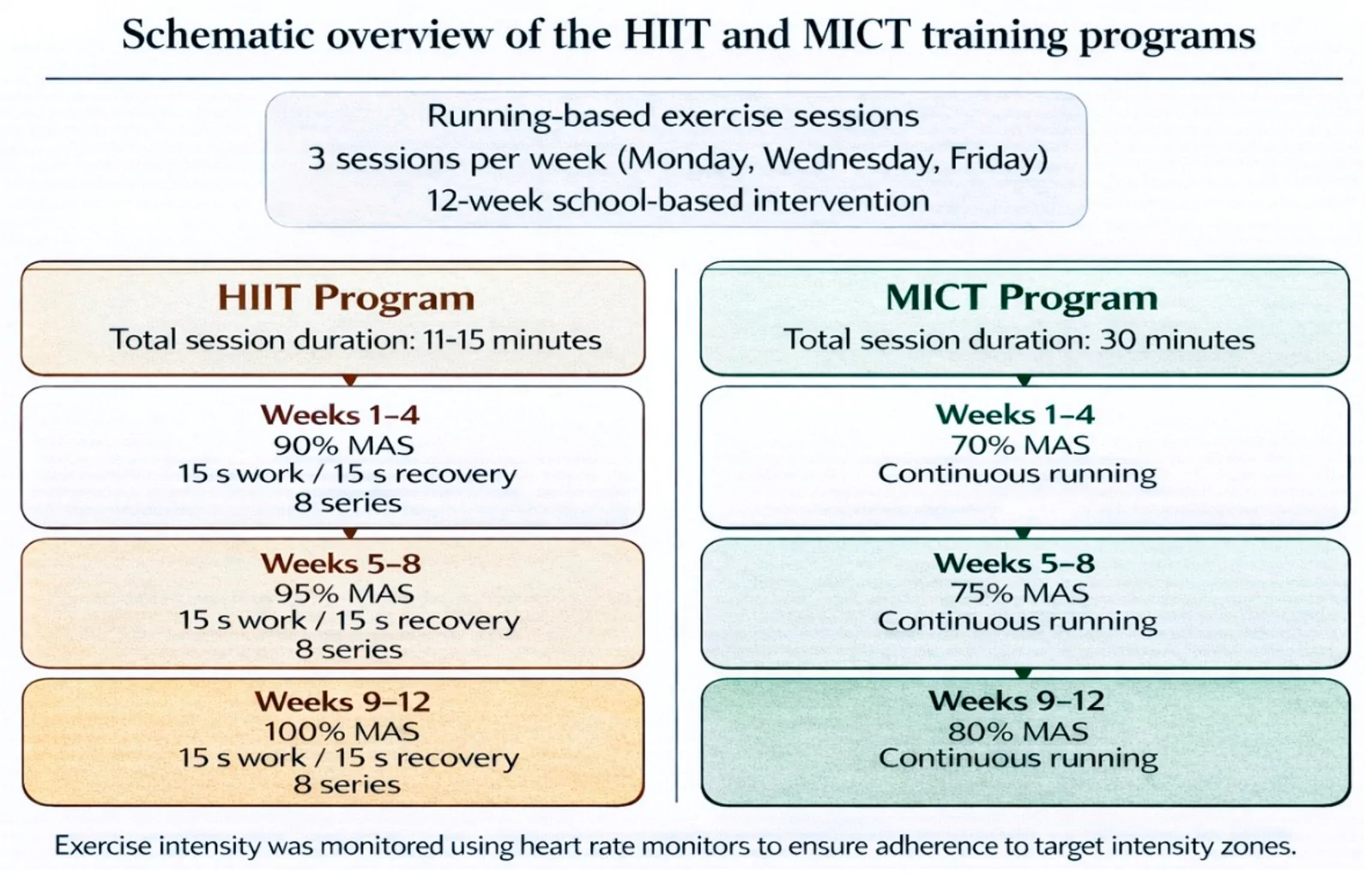
Training program schematic (12-week progression and total session duration). MAS: Maximal Aerobic Speed.

The moderate-intensity continuous training (MICT) program was also progressively structured over the 12 weeks. Exercise intensity increased from 70% MAS during weeks 1–4, to 75% MAS in weeks 5–8, and 80% MAS during weeks 9–12. Participants completed a single continuous bout per session, with a work-to-recovery structure of 30 min of exercise followed by 10 min of recovery in the first two phases, and 25 min of exercise followed by 10 min of recovery in the final phase. The total duration of MICT sessions ranged from 30 to 40 min throughout the intervention period. The schematic overview of the HIIT and MICT training protocols implemented during the 12-week intervention is presented in Figure 2.

### 2.6. Measures

Anthropometric measurements included body weight, height and body mass index (BMI). Body weight was measured to the nearest 0.1 kg using a calibrated electronic scale (Terraillon TPRO 3100, Terraillon SAS, Croissy-sur-Seine, France), and height was assessed to the nearest 0.1 cm with a wall-mounted stadiometer (SECA 216, SECA GmbH & Co., KG, Hamburg, Germany), in accordance with the World Health Organization (WHO) standard protocol. BMI was calculated as weight divided by height squared (kg/m^2^).

Blood pressure and heart rate were measured before and after 12 weeks of physical training (HIIT and MICT). Before the start of the measurements, each participant remained in a seated position for 10 min with a back support. Systolic blood pressure (SBP), diastolic blood pressure (DBP), and heart rate were measured according to validated standard procedures for the use of a manual sphygmomanometer [9]. respect and comply with the recommendations for blood pressure measurement [21,22,23]. using digital arm blood pressure monitors (Bosch + Sohn, GmbH u.Co., KG, Jungingen, Germany). The recorded values are the mean of the three measurements.

Insulin resistance was assessed using the Homeostasis Model Assessment of Insulin Resistance (HOMA-IR) index, which is based on fasting glucose and fasting insulin concentrations and calculated according to the formula proposed by [24]. HOMA-IR = [fasting insulin (µU/mL) × fasting glucose (mmol/L)] ÷ 22.5.

In accordance with established reference data in the Korean population, a threshold of 2.2 was used to categorize participants into two groups: HOMA-IR < 2.2 and HOMA-IR ≥ 2.2 at baseline [24].

Physical fitness was assessed prior to the intervention using the VAM-Eval incremental field test to determine each participant’s individual Maximal Aerobic Speed (MAS). This measure was used exclusively to calibrate and individualize the running intensities prescribed for both the HIIT (90–100% MAS) and MICT (70–80% MAS) protocols. Physical fitness variables were not included as primary outcomes of the present study, which was specifically designed to investigate the effects of exercise training on cardiometabolic and insulin resistance markers. The physical fitness data collected within this broader research project are currently being analyzed and will be reported in a forthcoming companion manuscript.

### 2.7. Statistical Analysis

All statistical analyses were performed using SPSS version 22 (IBM Corp., Armonk, NY, USA). Continuous variables were expressed as mean ± standard deviation (SD). Baseline comparisons between intervention groups were conducted using one-way analysis of variance (ANOVA) for continuous variables.

The primary outcome was post-intervention insulin resistance assessed by the HOMA-IR index. Group differences in post-intervention HOMA-IR were examined using linear regression models following an analysis of covariance (ANCOVA) approach, with baseline HOMA-IR included as a covariate. Regression coefficients (β) and 95% confidence intervals (CIs) were reported.

Three hierarchical models were constructed: an unadjusted model (M0); a partially adjusted model (Model I) adjusted for age and sex; and a fully adjusted model (Model II) further adjusted for baseline body mass index (BMI) and baseline HOMA-IR.

Stratified analyses were additionally performed according to baseline weight status (overweight and obesity) to explore potential effect modification by BMI category. All statistical tests were two-tailed, and statistical significance was set at *p* < 0.05.

## 3. Results

### 3.1. Characteristics of Participants

The baseline characteristics of the study population are presented in Table 1. A total of 90 adolescents were included, with 30 participants (33.3%) allocated to each group: control, MICT and HIIT. The overall mean age of participants was 14.9 ± 1.8 years.

At baseline, statistically significant between-group differences were observed for age, body mass index (BMI), systolic blood pressure (SBP), and HOMA-IR (*p* < 0.05). No significant difference was found for diastolic blood pressure (DBP) between groups (*p* = 0.098).

### 3.2. Association Between Exercise Intervention Modality and Insulin Resistance (HOMA-IR)

Regression analyses showed a significant negative association between exercise intervention modality and insulin resistance (measured by HOMA-IR) compared with the control group (Table 2). In the overall sample, both MICT and HIIT were associated with significant reductions in HOMA-IR in the crude model (M0). These associations remained robust after partial (Model I) and full (Model II) adjustment (all *p* < 0.001). In the fully adjusted model (Model II), MICT was associated with a reduction in HOMA-IR of −0.773 (95% CI: −0.939, −0.607), while HIIT was associated with a greater reduction of −0.895 (95% CI: −1.068, −0.722).

Stratified analyses according to baseline weight status further supported these findings (Table 3). Among overweight adolescents (BMI < 28), both MICT and HIIT were associated with significant decreases in HOMA-IR across all models. In Model II, reductions of −0.896 (95% CI: −1.109, −0.682) and −0.995 (95% CI: −1.236, −0.755) were observed for MICT and HIIT, respectively (*p* < 0.001). Similarly, in obese adolescents (BMI ≥ 28), significant associations persisted after full adjustment, with HOMA-IR reductions of −0.506 (95% CI: −0.710, −0.302) for MICT and −0.839 (95% CI: −1.035, −0.644) for HIIT (*p* < 0.001).

### 3.3. Robustness of the Association Across Baseline Weight Status

To assess whether the association between exercise modality and insulin resistance differed by baseline weight status, stratified regression models were performed using BMI categories (overweight [BMI < 28] and obesity [BMI ≥ 28]) (Table 3). In adolescents with BMI < 28, both MICT and HIIT were associated with significant reductions in HOMA-IR compared with the control group in the fully adjusted model. Specifically, in Model II, MICT was associated with a reduction of −0.896 (95% CI: −1.109, −0.682) and HIIT with −0.995 (95% CI: −1.236, −0.755) (*p* < 0.001). For adolescents with BMI ≥ 28, reductions of −0.506 (95% CI: −0.710, −0.302) for MICT and −0.839 (95% CI: −1.035, −0.644) for HIIT were observed (*p* < 0.001). These results suggest that both exercise modalities have a positive effect on insulin resistance, independent of baseline weight status. The β coefficients remained largely consistent between the crude model (M0) and the fully adjusted model (Model II), indicating robustness of the associations and suggesting that the observed effects were not substantially driven by baseline differences between groups. In the obesity stratum, a modest attenuation of the β coefficients was observed between M0 and Model II (HIIT: −0.914 to −0.839; MICT: −0.538 to −0.506), consistent with the presence of mild positive confounding attributable to baseline differences in HOMA-IR and BMI; adjustment for these variables in Model II therefore provides a more accurate estimate of the independent exercise effect. Notably, for HIIT in the overweight stratum, the β coefficient increased in magnitude from M0 (β = −0.852) to Model II (β = −0.995), a pattern indicative of negative confounding, whereby lower baseline HOMA-IR values in the overweight subgroup partially masked the true effect of HIIT in the crude model; after adjustment, the independent effect of HIIT on HOMA-IR reduction is revealed to be stronger than the unadjusted estimate suggested. The consistency of statistical significance across all models (all *p* < 0.001) further confirms the robustness of these findings.

## 4. Discussion

A major finding of the present study is that participation in structured exercise interventions, whether HIIT or MICT, was associated with significant reductions in insulin resistance compared with the control group. These associations remained robust after adjustment for age, sex, baseline BMI, and baseline HOMA-IR, indicating that the observed improvements were primarily attributable to the training interventions rather than baseline differences between groups. The magnitude of the observed effects, with β coefficients ranging from approximately −0.77 to −0.90 in fully adjusted models, suggests clinically meaningful short-term improvements in insulin sensitivity. These findings are consistent with recent meta-analytic evidence in youths with excess weight [16,25]. reported that exercise interventions were associated with a mean reduction in HOMA-IR of −0.87, while [16,25]. found a weighted mean reduction of −0.82 in children and adolescents with overweight or obesity. Taken together, these data reinforce the clinical relevance of the improvements observed in the present trial.

When analyses were stratified according to baseline weight status, significant reductions in HOMA-IR were observed in both overweight and obese adolescents. Although effect sizes differed between BMI categories, the persistence of beneficial associations across strata suggests that in obese adolescents in insulin sensitivity may occur independently, or only partially dependently, of changes in body mass [12,26]. This observation is consistent with previous intervention studies and meta-analyses indicating that improvements in insulin sensitivity can be achieved even in the absence of substantial weight loss, particularly when exercise is performed regularly and at sufficient intensity [27,28].

The slightly greater reduction in HOMA-IR observed in the HIIT group compared with the MICT group aligns with findings from several recent meta-analyses, which have reported comparable or superior metabolic benefits of HIIT despite shorter session durations [13,18,29]. The higher metabolic demand associated with high-intensity exercise may result in more substantial insulin-dependent glucose uptake and enhanced activation of skeletal muscle pathways. This suggests that HIIT could offer advantages in terms of both metabolic improvements and time efficiency.

Several physiological mechanisms may explain the improvements in insulin resistance observed following both training modalities. Regular aerobic exercise has been shown to increase skeletal muscle GLUT4 expression, enhance insulin signaling pathways involving IRS-1 and Akt phosphorylation, improve mitochondrial oxidative capacity, and reduce systemic inflammation [14,24,30]. Additionally, exercise contributes to reductions in visceral adiposity, which plays a critical role in the pathogenesis of insulin resistance through mechanisms involving lipotoxicity, oxidative stress, and inflammatory signaling [31]. Together, these mechanisms provide a plausible biological basis for the reductions in HOMA-IR observed in the present intervention.

The findings of this study also align with large-scale evidence from pediatric populations. A recent meta-analysis including more than 3000 children and adolescents reported significant reductions in fasting insulin and HOMA-IR following exercise interventions, with greater benefits observed when physical activity was of moderate to vigorous intensity [16,25]. Similarly, another meta-analysis focusing on overweight and obese youth demonstrated improvements in glucose metabolism and insulin sensitivity following structured exercise programs, reinforcing the role of physical activity as a key non-pharmacological strategy in this population [13,16].

The present study provides additional evidence that insulin resistance, assessed using the HOMA-IR index, constitutes an important metabolic concern among overweight and obese Moroccan adolescents. In a national context marked by a steady increase in the prevalence of adolescent overweight and obesity, these findings are consistent with recent data reported in Morocco, where excess body weight has been observed in approximately 7 to 10% of adolescents for overweight and 3 to 5% for obesity, depending on the region [32]. Similar trends have been reported across several Maghreb countries, highlighting that the metabolic consequences of rapid lifestyle transitions are already evident in adolescence in this region [2,33].

Meta-analytic evidence has consistently shown that obese adolescents exhibit significantly higher fasting insulin levels and HOMA-IR values compared with their normal-weight peers, supporting the notion that obesity represents an early determinant of metabolic dysfunction [24,33]. Data from Tunisian and Algerian populations further corroborate these findings, indicating that elevated insulin resistance in overweight and obese adolescents is a regional phenomenon rather than an isolated observation [18,34].

The interpretation of insulin resistance during adolescence requires careful consideration of pubertal status. Puberty is characterized by physiological hormonal changes that induce a transient reduction in HIIT group compared with the MICT, even in healthy adolescents [5,28]. This physiological phenomenon may be exacerbated by excess adiposity, leading to higher HOMA-IR values during this developmental period. Recent studies have suggested that higher HOMA-IR thresholds, sometimes exceeding 3.0, may be required to accurately identify insulin resistance during puberty [34]. These findings highlight the importance of contextualizing HOMA-IR values according to age and developmental stage in order to avoid misclassification and inappropriate interpretation of metabolic risk in adolescent populations.

Beyond biochemical markers, lifestyle-related factors, including physical activity level, sedentary behavior, and physical fitness, play a central role in the modulation of insulin sensitivity [35]. Regular physical activity has been shown to enhance skeletal muscle glucose uptake, improve insulin signaling, and reduce low-grade systemic inflammation, thereby contributing to improved metabolic health [14,25]. In Moroccan adolescents, increasing exposure to sedentary behaviors, often driven by prolonged screen time and reduced engagement in structured physical activity, may partly explain the observed rise in insulin resistance and cardiometabolic risk.

Taken together, these results suggest that school-based exercise programs incorporating either MICT or HIIT can induce favorable short-term metabolic adaptations in overweight and obese adolescents While the relatively short duration of the intervention limits conclusions regarding the long-term persistence of these effects, the present findings underscore the importance of early interventions [13]. Future studies should focus on longer-term follow-up to assess the sustainability of metabolic improvements and the role of physical activity in delaying the onset of chronic conditions such as type 2 diabetes or cardiovascular disease in adolescents.

An additional strength of the present intervention is its school-based delivery. This is important because feasibility and scalability are central when designing preventive strategies for adolescents. Previous evidence indicates that HIIT is a feasible and time-efficient approach in youth, and school-based low-volume HIIT has already shown acceptable attendance and beneficial cardiometabolic effects in adolescents. Therefore, the present findings are not only biologically relevant but also practically transferable to real-world school settings.

### 4.1. Practical Implications

The present findings suggest that school-based exercise interventions can be feasibly implemented to improve insulin sensitivity in overweight and obese adolescents. Both high-intensity interval training and moderate-intensity continuous training were effective, indicating that schools may flexibly choose the modality that best fits time constraints, facilities, and pedagogical objectives. Notably, the short duration of HIIT sessions may represent a practical advantage in school settings with limited curriculum time, while still providing meaningful metabolic benefits. These results support the integration of structured, supervised exercise programs into regular physical education classes as a preventive strategy to reduce early metabolic risk during adolescence. Beyond aerobic-based modalities, it is important to acknowledge that the optimal exercise prescription for managing obesity may vary across the lifespan. Emerging approaches such as resistance training, concurrent training, and Integrative Neuromuscular Training (INT), a structured modality targeting both health-related (cardiorespiratory fitness, muscular strength, flexibility) and skill-related (balance, motor coordination, postural control) components of physical fitness, have shown promising outcomes in individuals with obesity across different age groups, from children to older adults [36]. Practitioners and physical education teachers working with youth populations should therefore consider a multimodal exercise approach that addresses not only metabolic outcomes but also motor competence and musculoskeletal health, particularly given the postural and biomechanical challenges associated with pediatric obesity.

### 4.2. Limitations

This study has several limitations that should be considered when interpreting the findings. First, the relatively small sample size may limit statistical power and restrict the generalizability of the results to the broader population of Moroccan adolescents. The short duration of the intervention (12 weeks) prevents conclusions about the long-term sustainability of the observed metabolic improvements. Future studies should incorporate longer follow-up periods and post-intervention assessments to evaluate whether the exercise-induced improvements in insulin resistance are maintained after cessation of the structured program. Additionally, while dietary intake is a key determinant of insulin resistance, it was not strictly controlled, and reliance on self-reported data introduces the potential for recall bias. The use of the HOMA-IR index, although widely used, may not fully capture insulin resistance compared to the hyperinsulinemic-euglycemic clamp, which was not feasible in this school-based setting. Moreover, measurements of biochemical and anthropometric parameters may have been influenced by factors such as circadian rhythms, hydration status, pubertal development, and the school environment. Finally, physical activity outside of the structured training sessions and other lifestyle factors such as sleep and sedentary behavior were not objectively monitored, which could have introduced variability in metabolic outcomes. A further limitation of this study is the absence of physical fitness outcomes as primary endpoints. Although the VAM-Eval test was conducted to determine individual Maximal Aerobic Speed for training load calibration, broader fitness assessments (e.g., cardiorespiratory fitness, muscular strength) were not analyzed in this manuscript. These data are currently being prepared for a companion publication, allowing the present study to maintain its focused investigation of cardiometabolic and insulin resistance markers. These limitations should be considered when interpreting the results and before extrapolating the findings to larger or more diverse adolescent populations.

## 5. Conclusions

Both HIIT and MICT were shown to significantly reduce markers of insulin resistance in overweight and obese non-diabetic Moroccan adolescents, with a slightly greater effect observed for HIIT, although this difference was not statistically significant. These findings are in line with current evidence supporting the effectiveness of structured exercise programs for improving glucose regulation in overweight and obese adolescents, and support the integration of structured exercise programs into school-based physical education as a non-pharmacological strategy to improve insulin sensitivity and limit early metabolic risk. Future research should focus on longer-term interventions with objective dietary and lifestyle monitoring, larger and more diverse samples, and follow-up assessments to determine the durability and broader applicability of these findings.

## Figures and Tables

**Table 1:** Baseline characteristics of overweight and obese adolescents according to intervention group.

Variable	Control (*n* = 30)	MICT (*n* = 30)	HIIT (*n* = 30)	*p*-Value
Age (years)	15.0 ± 1.63	14.5 ± 1.80	15.2 ± 1.44	0.045
BMI (kg/m^2^)	27.38 ± 1.31	26.32 ± 1.57	26.32 ± 1.80	0.013 *
SBP (mmHg)	124.03 ± 4.95	118.97 ± 4.12	122.73 ± 3.49	<0.001 ***
DBP (mmHg)	80.63 ± 5.03	78.53 ± 3.10	80.10 ± 3.19	0.098
HOMA-IR	2.72 ± 0.30	1.88 ± 0.51	1.86 ± 0.42	<0.001 ***

Values are presented as mean ± standard deviation. *p*-values refer to between-group comparisons at baseline (ANOVA). BMI: body mass index; SBP: systolic blood pressure; DBP: diastolic blood pressure; HOMA-IR: homeostasis model assessment of insulin resistance. * *p* < 0.05; *** *p* < 0.001.

**Table 2:** Association between exercise intervention (MICT and HIIT) and insulin resistance outcomes compared with the control group. Values are presented as β coefficients with 95% confidence intervals (CIs).

Group	M0: Crude Model β (95% CI)	*p*-Value	Model I β (95% CI)	*p*-Value	Model II β (95% CI)	*p*-Value
Control	Reference	—	Reference	—	Reference	—
MICT	−0.847 (−1.061, −0.632)	<0.001	−0.778 (−0.938, −0.619)	<0.001	−0.773 (−0.939, −0.607)	<0.001
HIIT	−0.867 (−1.081, −0.652)	<0.001	−0.903 (−1.062, −0.745)	<0.001	−0.895 (−1.068, −0.722)	<0.001

**Table 3:** Association between exercise intervention (MICT and HIIT) and insulin resistance stratified by baseline body mass index category. Values are presented as β coefficients with 95% confidence intervals (CIs).

BMI Category	Group	M0 β (95% CI)	*p*-Value	Model I β (95% CI)	*p*-Value	Model II β (95% CI)	*p*-Value
**Overweight**	**Control**	**Reference**	**—**	**Reference**	**—**	**Reference**	**—**
	MICT	−0.910 (−1.149, −0.672)	<0.001	−0.836 (−1.025, −0.647)	<0.001	−0.896 (−1.109, −0.682)	<0.001
	HIIT	−0.852 (−1.094, −0.611)	<0.001	−0.908 (−1.099, −0.716)	<0.001	−0.995 (−1.236, −0.755)	<0.001
**Obesity**	**Control**	**Reference**	**—**	**Reference**	**—**	**Reference**	**—**
	MICT	−0.538 (−0.770, −0.306)	<0.001	−0.491 (−0.684, −0.299)	<0.001	−0.506 (−0.710, −0.302)	<0.001
	HIIT	−0.914 (−1.137, −0.691)	<0.001	−0.839 (−1.030, −0.649)	<0.001	−0.839 (−1.035, −0.644)	<0.001

## Data Availability

The data presented in this study are available on request from the corresponding author. The data are not publicly available due to privacy and ethical restrictions.

## Abbreviations

The following abbreviations are used in this manuscript:

HIIT	High-intensity interval training
MICT	Moderate-intensity continuous training
MAS	Maximal Aerobic Speed
